# Scalable Correlated Local Approaches for Computing Valence and Core-Level Ionization Energies in Large Molecules

**DOI:** 10.1021/acs.jctc.6c00634

**Published:** 2026-06-24

**Authors:** Dávid Mester, Mihály Kállay

**Affiliations:** † Department of Physical Chemistry and Materials Science, Faculty of Chemical Technology and Biotechnology, Budapest University of Technology and Economics, Műegyetem rkp. 3., H-1111 Budapest, Hungary; ‡ HUN-REN-BME Quantum Chemistry Research Group, Műegyetem rkp. 3., H-1111 Budapest, Hungary; § MTA-BME Lendület Quantum Chemistry Research Group, Műegyetem rkp. 3., H-1111 Budapest, Hungary

## Abstract

A scalable framework is introduced for the calculation of valence and core ionization energies within the second-order algebraic-diagrammatic construction [ADC(2)] formalism. The approach is based on the construction of state-specific orbital domains that are determined entirely by the underlying electronic structure and ionization process. As a result, the procedure adapts automatically to the character of the ionized state and can be applied in a genuine black-box manner without system-specific tuning. The methodology is tested for conventional ADC(2), its spin-opposite-scaled variant, and an ADC(2)-based double-hybrid functional. Benchmark calculations show that the errors introduced by the local approximation remain far below the intrinsic uncertainties of the underlying correlated methods. For both valence and core ionization energies, the deviations are typically on the order of a few hundredths of an electronvolt. At the same time, substantial reductions of the orbital space are achieved, leading to the significant acceleration of the most expensive steps of the correlated treatment. The efficiency and robustness of the approach are demonstrated for extended molecular systems of practical relevance. Once the reference orbital set is obtained, the valence ionization energy of a 132-atom thermally activated delayed fluorescence emitter can be determined within approximately 20 min using a triple-ζ basis set, while the 4 N *K*-edge core ionization energies of a 372-atom porphyrin derivative are obtained within about 2 h. In both cases, the corresponding ADC(2) eigenvalue problem itself requires only about 1 min per state. The proposed framework therefore enables routine applications of ADC(2)-based methods to molecular systems that are beyond the reach of conventional implementations.

## Graphical abstract

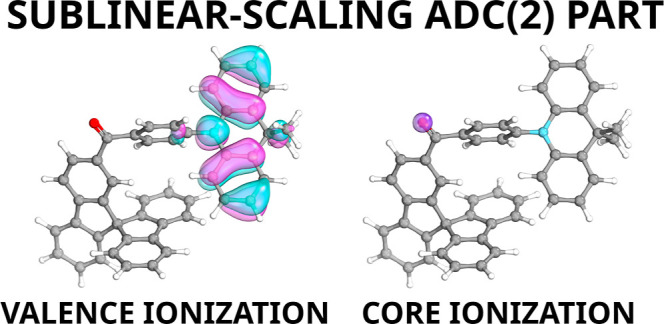

## 1 Introduction

The accurate determination of ionization energies provides a fundamental energetic reference for a broad range of technologically and biologically relevant processes.^1–6^ As the minimum energy required for electron removal, the ionization potential (IP) controls the onset of charge formation and shapes the subsequent response of a molecular system under an electrical bias, photoexcitation, or ionizing radiation. Consequently, quantitatively reliable ionization energy values are essential for predictive materials design and for a consistent mechanistic description of radiation and photoinduced processes.

Despite their importance, the reliable computation of ionization energies for extended molecular systems remains a significant challenge. Density functional theory (DFT) offers a computationally efficient route, but ionization energies are most commonly approximated as the negative of the corresponding Kohn–Sham (KS) orbital energies, effectively describing the process as a simple one-electron removal from a single determinant.^7,8^ In this picture, the final state is represented by a single ionized configuration, and relaxation and correlation effects associated with the response of the remaining electrons are neglected. More advanced DFT-based approaches aim to overcome these limitations by explicitly accounting for orbital relaxation. In orbital-optimized methods,^9–13^ the electronic structure is variationally relaxed for each ionized state, thereby providing a more consistent description of the final state. However, such approaches often suffer from practical difficulties, including convergence issues, variational collapse, and the need for careful control of the electronic configuration. Alternative strategies, such as the Slater transition concept, employ fractional occupations to approximate relaxation effects in a computationally efficient manner.^14^

Wave function-based methods, such as the algebraic-diagrammatic construction (ADC)^15^ and equation-of-motion coupled-cluster (EOM-CC)^16–18^ approaches, provide a more rigorous treatment of electron correlation and orbital relaxation effects and have become the methods of choice for accurate ionization energy calculations. In the EOM-CC framework, ionized states are obtained by diagonalizing a similarity-transformed Hamiltonian in a manifold of (*n* – 1)-electron configurations,^19–22^ whereas in the ADC formalism an effective Hermitian matrix is constructed from a diagrammatic perturbation expansion of the polarization propagator and diagonalized in a corresponding excitation manifold.^23–27^ In both cases, relaxation effects are incorporated through couplings to higher excited configurations. In these approaches, ionization processes are naturally described in terms of Dyson orbitals,^28^ which provide a physically consistent representation of the ionized state beyond a single-orbital picture. However, their steep computational scaling with system size severely limits their applicability to large molecular systems of practical interest.

For core ionizations, the situation is further complicated by the high energetic position of the corresponding states. In practice, the core–valence separation (CVS) approximation is commonly employed to restrict the calculation to the relevant subspace, thereby enabling efficient access to core-ionized states. This can be achieved either by neglecting the corresponding molecular integrals^29^ or by projecting out the coupling elements after their evaluation.^30^ While these approaches are straightforward to implement, they often involve the computation of unnecessary quantities and therefore do not fully eliminate the associated computational overhead. As a result, the overall calculation remains demanding due to the underlying complexity.

An important alternative class of approaches for the calculation of ionization energies is provided by many-body Green’s-function methods, in particular the *GW* approximation.^31,32^ In contrast to ADC and EOM-CC, which describe ionized states through explicit configuration spaces, *GW* methods determine quasiparticle energies from a frequency-dependent self-energy constructed from the one-particle Green’s function and the dynamically screened Coulomb interaction. As a result, the main physical emphasis of *GW* is on electronic screening, with practical variants such as *G*_0_ *W*_0_,^33^ eigenvalue-self-consistent *GW*,^34^ and quasiparticle-self-consistent *GW* ^35^ differing in the level of self-consistency used to refine the quasiparticle description. These features make *GW* methods highly attractive for extended systems and valence quasiparticle spectra, while their performance for localized core ionizations depends sensitively on the selected reference, orbital relaxation, and the treatment of the screened interaction.

The increasing demand for accurate ionization energies in large molecular systems has therefore stimulated the development of more efficient computational strategies. On the one hand, considerable effort has been devoted to improving the efficiency of existing implementations.^36–42^ On the other hand, the development of lower cost yet accurate methods, such as double-hybrid (DH) density functionals, provides an appealing alternative.^43,44^ A further promising direction is the exploitation of physically motivated orbital representations, in which only those orbitals that significantly contribute to the correlated treatment are retained.^45–48^ Such approaches aim to reduce the effective size of the problem while preserving the essential physics of the ionization process. This, in turn, leads to a substantial reduction of the cost of the correlated calculations. Closely related developments exploit the local character of electronic transitions to formulate reduced-scaling excited-state methods; however, these approaches have been predominantly designed for excitation energies^49–60^ and have only limited applicability to ionization problems.^61^ In addition, independently of these approaches, recent efforts have focused on extending promising ionization methods to systems with a pronounced multireference character, further broadening their scope.^40,62–66^

The present work targets a complementary regime within this broader methodological landscape. Second-order ADC [ADC(2)]-based^67,68^ IP methods are derived from a perturbative expansion of the polarization propagator and yield a second-order, Hermitian effective eigenvalue problem in which relaxation and correlation effects arise through the coupling of the one-hole (1h) and two-hole–one-particle (2h1p) manifolds. In contrast, higher order EOM-CC methods provide a more systematically improvable, albeit substantially more expensive, framework, whereas *GW* methods provide a different balance by emphasizing dynamical screening and ring-type contributions. The aim of the present approach is therefore not to replace EOM-CC or *GW*, but to extend ADC(2)-based valence and core ionization calculations, with their outstanding accuracy-to-cost ratio, to molecular systems that are too large for conventional canonical treatments.

In this work, we present a scalable framework for the calculation of valence and core ionization energies within the ADC(2) formalism based on state-specific orbital domains. The approach exploits the local nature of the electronic response to ionization and enables a substantial reduction of the correlated orbital space while preserving the accuracy of the underlying method. The resulting procedure is fully automatic and can be applied in a black box manner, making it particularly suitable for large and chemically diverse molecular systems. Its performance is demonstrated for representative applications ranging from functional organic materials to large-scale systems that are relevant for spectroscopy.

## 2 Theory

### 2.1 Valence- and Core-Level Ionization at the ADC(2) Level

IP-ADC­(2) can be naturally interpreted in a manifold picture. The basic idea is to describe an (*n* – 1)-electron state arising from the removal of an electron from an occupied orbital in an interacting system. The resulting final state is generally not well described by a single 1h configuration but instead involves coupling to more complex ionization classes, most prominently the 2h1p manifold that accounts for correlation and orbital relaxation of the remaining electrons. At the ADC(2) level, this coupling is incorporated consistently to second order, leading to a systematic refinement of the primary ionization lines through correlated corrections, while at the same time allowing for the appearance of satellite features arising from electron correlation.

Starting from a Hartree–Fock (HF) reference determinant Ψ_0_, a generic ionization operator is introduced spanning the singly ionized space: ${Ĉ}_{1}=\sum_{i}c_{i}i^{-}$ and the (*n* – 1)-electron wave function reads $\Psi_{n-1}={Ĉ}_{1}\Psi_{0}=\sum_{i}c_{i}\Phi_{i}$ , where *i* ^–^ annihilates an electron in occupied spatial orbital *i* and Φ_*i*_ denotes the corresponding 1h determinant. Projecting the electronic Hamiltonian onto this subspace leads to the Koopmans-like IP-configuration interaction singles (IP–CIS) equations. While Koopmans’ theorem may be viewed as a zeroth-order description of ionization, and IP-CIS formally constitutes a first-order treatment, the two become equivalent in a canonical HF basis. In this case, the resulting ionization energies (ω_*k*_^IP‑CIS^) reduce to the negative of the occupied orbital energies (ε_*k*_): ω_*k*_^IP‑CIS^ = −ε_*k*_ and the corresponding eigenstates describe pure orbital removals. Accordingly, the eigenvectors are orthonormal unit vectors aligned with the individual 1h configurations, reflecting the absence of configurational mixing within the 1h space at this level: Ψ_*n*–1_ = Φ_*k*_. While this starting point is useful conceptually, it neglects the fact that an ionized system can lower its energy by relaxing and correlating the remaining electrons in response to the created hole.

At the second order, the leading relaxation and correlation effects are associated with the interaction between the 1h sector and the 2h1p manifold, i.e., configurations in which, in addition to the primary hole, a second hole is accompanied by a particle excitation that redistributes the remaining electron density. Introducing the corresponding operator, ${Ĉ}_{2}=\sum_{ija}c_{ia,j}a^{+}i^{-}j^{-}$ , where *a* denotes a virtual orbital, the double excitation coefficients *c*_*ia*,*j*_ quantify the extent to which 2h1p configurations contribute to the final state. At the second order, their form is determined by interaction matrix elements connecting the 1h and 2h1p sectors, as well as by the corresponding orbital-energy differences^44,69^

1 $$c_{ia,j}=\frac{\langle \Phi_{ij}^{a}|\mathrm{V̂}|{Ĉ}_{1}\Phi_{0}\rangle }{\varepsilon_{i}+\varepsilon_{j}-\varepsilon_{a}+\omega_{k}}=\frac{\langle \Phi_{ij}^{a}|\mathrm{V̂}|\Phi_{k}\rangle }{\varepsilon_{i}+\varepsilon_{j}-\varepsilon_{a}+\omega_{k}}$$

where |Φ_*ij*_^*a*^⟩ stands for a 2h1p determinant. This expression has a clear physical interpretation. The numerator contains interaction matrix elements through which the presence of the primary hole gives rise to additional electronic rearrangements associated with the relaxation and screening of the ionized state. The denominator reflects the orbital-energy offsets that govern the relative energetic accessibility of the corresponding 2h1p configurations—i.e., how energetically resonant they are with the primary ionized state—and thus their weight in the final wave function.

In practical IP-ADC(2) implementations, the influence of the 2h1p configurations is incorporated into an effective eigenvalue problem acting within the 1h space. This leads to a nonlinear eigenvalue equation

2 $${Ã}^{IP‐ADC\left(2\right)}\left(\omega^{IP‐ADC\left(2\right)}\right)c=\omega^{IP‐ADC\left(2\right)}c$$

where ${Ã}^{IP‐ADC\left(2\right)}$ denotes the so-called effective ADC(2) Jacobian and ω^IP–ADC(2)^ is the corresponding ionization energy. Importantly, the resulting ionized states are not limited to pure orbital removals. Since the eigenvectors in IP-ADC(2) are no longer orthonormal unit vectors, the final state is expanded—already at the level of the effective 1h representation—as Φ_*n*–1_ = ∑_i_c_i_ Φ_*i*_ ≠ Φ_*k*_. This allows multiple ionization channels to mix, while the satellite character reflects the effective influence of the underlying 2h1p configurations. In this sense, IP-ADC(2) provides a consistent second-order description of the electron-removal problem in which relaxation and correlation effects enter through the coupling between the 1h and 2h1p sectors.

Core binding energies correspond to ionizations from deeply bound core orbitals and, therefore, are located at large ionization energies. A direct application of iterative eigensolvers to eq 2 would be inefficient because the algorithms naturally converge the lowest IP roots first, which correspond to valence ionizations. The CVS approximation addresses this issue by neglecting couplings between configurations involving core holes and those containing only valence excitations.^29^ As a result, the ADC(2) secular matrix acquires an approximate block structure that enables a direct solution for the core-ionized states. This approximation is physically justified by the energetic and spatial separation of core and valence orbitals: excitations involving core electrons are typically well separated in energy from valence excitations, and the corresponding coupling matrix elements that would mix these sectors are small. In practice, this amounts to neglecting classes of two-electron interactions that would otherwise couple core and valence excitation manifolds, thereby restricting the problem to a reduced space that retains the dominant physics of core-ionization.

To formulate CVS, the occupied orbital space is partitioned into an active (*I*) subset and the remaining inactive (*i*) occupied orbitals. The active orbitals typically correspond to the set of core orbitals from which the ionization occurs, while the inactive orbitals comprise all other correlated occupied orbitals. The CVS restriction is introduced already at the 1h level by limiting the ionization operator to core-hole configurations, ${Ĉ}_{1}=\sum_{I}c_{I}I^{-}$ , and the associated 2h1p manifold is restricted consistently to configurations that retain a core hole, i.e., those in which one of the hole indices corresponds to an active core orbital, while the second hole resides in the inactive space: ${Ĉ}_{2}=\sum_{iJa}c_{ia,J}a^{+}i^{-}J^{-}$ . From the secular-matrix perspective, this corresponds to neglecting couplings that mix core-hole and purely valence-hole sectors, thereby yielding a reduced eigenvalue problem that targets the core-ionized states directly. Importantly, while this restriction removes interactions that mix core and valence excitation spaces, it retains the leading relaxation and polarization effects of the valence electrons in response to the core hole through the coupling between core-hole 1h and the corresponding 2h1p configurations. Additional details of our IP-ADC(2) implementation can be found in ref 44, while the CVS-IP-ADC(2) implementation is discussed in detail in ref 43.

### 2.2 Local Approximations

The representation of ionized states using canonical molecular orbitals (MOs) leads to steep computational scaling, even when the underlying electronic response is spatially localized. In realistic molecular systems, ionizations are typically governed by a limited subset of orbitals associated with chemically confined regions, such as chromophores, functional groups, or other localized motifs. Although the canonical representation may distribute the response over the entire orbital space, the accompanying relaxation and polarization processes remain inherently localized. This provides the conceptual foundation for reduced-scaling approaches: the number of orbitals required for an adequate description of a given ionized state does not grow extensively with the system size, provided that an appropriate orbital subspace is identified.

Ionization is not described only by the removal of an electron from a single occupied orbital. The creation of a hole induces a correlated response of the remaining electrons, leading to relaxation and polarization that stabilizes the final state. Consequently, a reliable local approximation must account not only for the orbitals directly involved in the ionization process but also for those required to describe the environmental response.^70,71^

Locality emerges most naturally in a representation based on spatially compact orbitals,^72^ where correlation effects decay with the distance and the dominant contributions to the final wave function originate from a confined region surrounding the hole. This motivates the construction of state-specific orbital domains that are sufficiently compact to enable reduced-scaling treatments while remaining complete enough to capture the relaxation and correlation effects. To this end, we build upon our previously developed domain construction framework for electronic excitations.^60^ Ionizations, however, constitute formally simpler processes, allowing a streamlined version of the algorithm to be employed, with the only conceptual difference being that the excited virtual orbital is formally replaced by a continuum orbital.^73,74^

For a given IP state, a state-specific domain is constructed to reflect the local nature of the electronic response. As a practical starting point, a low-cost approximate description of the ionization is obtained from a formal IP-CIS calculation. In this case, the eigenstate coincides with the corresponding 1h determinant, so identifying the dominant ionization channel does not require any additional step. Although this level of theory lacks electron correlation, it reliably identifies the occupied orbital defining the location of the hole. Rather than treating this orbital in its canonical, fully delocalized form, the description is recast in terms of localized MOs (LMOs).^72^ The LMOs that collectively span the canonical hole orbital are selected into a so-called strict domain by analyzing its expansion in the occupied LMO basis. The LMOs are ranked by the magnitude of their expansion coefficients, and LMOs are included successively until their cumulative contribution, that is, the sum of the corresponding expansion coefficients, reaches a predefined completeness threshold, denoted by *T*_LMO_, thereby defining the minimal localized orbital space required to represent the hole.

However, the creation of a hole induces relaxation and polarization in its immediate chemical environment. To capture this response, the domain is extended to include LMOs that are spatially connected to the hole region. This extension is guided by Boughton–Pulay (BP) atom lists:^75^ any LMO whose loose BP atom list—defined using the parameter *T*_BPol_—shares at least one atom with that of a strict LMO is included. The orbitals added in this step form the environment LMO set, and together with the strict LMOs define the final LMO set. The virtual space is defined consistently by allowing polarization on the atoms associated with the hole. This is achieved by including projected atomic orbitals (PAOs) centered on atoms belonging to the union of the loose BP atom lists of a chosen LMO set, such as the strict or the final set, thereby forming the final PAO set.^70^ This reflects the physical expectation that the dominant response arises from the local charge redistribution near the ionization site.

The resulting domain thus contains three essential components: orbitals directly representing the hole, orbitals describing its local environment, and orbitals required for balanced correlation treatment. Once established, the atomic orbital (AO) and auxiliary basis functions required for the density-fitting approximation are also restricted to the spatial region defined by the final LMO set using atom lists obtained from their tight (defined by *T*_BPot_) and loose BP domains, respectively. This reduces the computational demand associated with the molecular integral evaluation and transformation. The occupied and virtual orbitals are subsequently reorthogonalized and canonicalized within the truncated AO basis to define the reduced space for the final (CVS-)­IP-ADC(2) calculation.^76,77^ Through this construction, the ionized-state problem is reformulated in a compact, system-specific orbital space that retains the essential physics of charge removal, relaxation, and correlation while enabling efficient treatments of large molecular systems by allowing the sublinear scaling of the correlation calculation with the presented algorithm.

### 2.3 General Considerations

From a computational perspective, the cost of IP-ADC(2) calculations is governed by two conceptually distinct steps. First, the ground-state second-order Møller–Plesset (MP2) amplitudes must be constructed and contracted with the corresponding integrals, which formally constitutes the rate-determining step of the calculation. This contribution scales noniteratively as $O\left(N^{5}\right)$ with the system size and represents the dominant computational bottleneck in conventional IP-ADC(2) calculations. Once the MP2-related intermediates are available, the ionization problem itself is solved through an iterative procedure involving the effective ADC(2) Jacobian. The matrix–vector products required in this step scale as $O\left(N^{4}\right)$ and must be carried out for each targeted ionized state. Consequently, the overall computational effort for valence IP calculations is dominated by the MP2 calculation, followed by the iterative solution of the corresponding eigenvalue problem.

The scaling behavior differs markedly for CVS-IP-ADC(2). Due to the CVS restriction, the ground-state MP2 contributions are completely eliminated, and the expensive $O\left(N^{5}\right)$ step is avoided. The remaining CVS-IP eigenvalue problem is reduced to operations that scale practically as $O\left(N^{3}\right)$ , with a mild prefactor that depends on the number of active occupied orbitals in the core space. As a result, the HF self-consistent field (SCF) procedure becomes the rate-determining step of CVS-IP-ADC(2), while molecular integral evaluation and transformation remain a notable, though secondary, contribution to the overall computational cost.

These different scaling characteristics have direct implications for the design of state-specific domains. For valence ionizations, substantial computational savings can be achieved if the size of the correlated orbital space is reduced more aggressively, as this enables a significant reduction of the $O\left(N^{5}\right)$ MP2 step. In contrast, for CVS-IP calculations the primary objective is not to accelerate the correlation treatment—which is already inexpensive—but rather to obtain meaningful speedups through more efficient integral evaluation and transformation. In this case, a computational gain can already arise solely from the fact that the selected orbitals are expanded only on a limited set of atoms. Even in the limiting case where the final domain formally spans the entire molecule—that is, includes all orbitals and atoms—the framework still provides a computational advantage. Although no orbitals are removed, each orbital is effectively confined to a restricted atomic region, which reduces the cost of integral evaluation and transformation and thereby accelerates the overall procedure. Consequently, state-specific domains can be constructed more conservatively for core ionizations, whereas stronger truncations are required for valence ionizations in order to realize substantial efficiency gains.

In addition to their computational differences, valence and core ionizations also differ markedly in their physical character. While a core hole is inherently confined to a single atomic center, valence ionization—particularly when involving a delocalized orbital—may extend over a substantial portion of the molecule. As expected from numerical experience, the domain construction scheme previously developed for valence excitations can be transferred to the description of valence ionizations without modification, with the only conceptual difference being that the virtual orbital involved in the excitation is formally replaced by a continuum orbital. Accordingly, we employ the previously established default parameters, namely *T*_LMO_ = 0.999, *T*_BPol_ = 0.95, and *T*_BPot_ = 0.9999. The first parameter ensures that the hole is accurately represented in the localized basis. The second, loose BP criterion guarantees that all relevant LMOs from the immediate environment of the ionization site are included in the final set, while also providing a sufficient number of auxiliary basis functions for reliable integral fitting. The third parameter ensures an adequate representation of the AO space. Furthermore, in analogy with the excited-state algorithm, the selection of PAOs for the virtual space can be based solely on the BP atom list of the strict LMO set.

For CVS-IP calculations, however, a somewhat-modified strategy is required. In this case, the strict LMO set typically consists of a single orbital localized on a single atom. As a result, applying the original loose BP criterion would lead to a final set containing only occupied LMOs centered at that atom. To avoid this overly restrictive description, a tighter BP criterion should be employed. Our numerical tests indicate that using *T*_BPol_ = *T*_BPot_ = 0.9999 provides a balanced description in which the local environment of the core hole is adequately represented. This choice ensures that the final LMO set spans not only the space of the AOs of the core-ionized atom but also that of a small number of neighboring atoms that participate in the relaxation response. A further modification concerns the construction of the PAO space. In the valence-IP framework, PAO selection is based on the strict LMO set. For core ionization, however, this would restrict the virtual space to PAOs centered on a single atom, which is insufficient to describe the polarization effects. Therefore, the PAO selection is instead performed using the BP atom list of the final LMO set, which incorporates the relevant local environment and provides a balanced description of the response to the core-hole.

## 3 Computational Details

### 3.1 Technical Details

All the calculations were carried out using the development version (26.1.0) of the MRCC suite of quantum chemical programs.^78,79^

All of the investigated methods are based on the ADC(2) formalism.^67,68^ In addition to the standard (CVS-)­IP-ADC(2) approach,^43,44^ the spin-opposite-scaling (SOS) variant was also considered.^80,81^ IP-SOS-ADC(2) significantly improves the accuracy of valence ionization energies relative to conventional IP-ADC(2);^74^ the precision is likewise enhanced, albeit to a lesser extent. In contrast, CVS-IP-SOS-ADC(2) leads to a noticeable deterioration in accuracy compared with standard CVS-IP-ADC(2) for core ionizations, although the precision of the results is markedly improved.^43^

Furthermore, we examined our ADC(2)-based DH time-dependent DFT ansatz,^82^ which has previously demonstrated an excellent performance for ionization processes.^43,44^ The success of this method stems from the more balanced description of the reference electronic structure provided by KS orbitals in combination with empirically scaled second-order correlation contributions. With the exception of the outstanding accuracy achieved by IP-SOS-ADC(2) for valence ionizations, the proposed SOS0-PBE0-2/(CVS-)­IP-ADC(2) functional^83^ outperforms its wave function-based counterparts in both accuracy and precision across all tested benchmarks for valence and core ionizations.^43,44^ We stress, however, that the purpose of this study is not to benchmark or rank these methods against one another. Rather, our objective is to demonstrate that the local approximations introduced here operate robustly and consistently across all of the considered ADC(2)-based variants. For convenience, the (CVS-)­IP prefix will be omitted from the names of the approaches in the text and figures hereinafter.

In this study, correlation-consistent triple-ζ basis sets were employed as AO basis sets. For valence ionizations, the cc-pVTZ basis sets^84^ were used, whereas for core ionizations the core–valence counterpart recontracted for exact two-component (X2C) relativistic calculations, cc-pCVTZ-X2C,^85,86^ was applied. The density fitting approximation was employed at both the HF/KS and post-HF/KS levels. For this purpose, the corresponding auxiliary basis sets proposed by Weigend and co-workers^87,88^ were employed whenever available; otherwise, those were generated using the AutoAux algorithm.^89^ The frozen-core approximation was applied in all post-HF/KS calculations. For the DFT contributions, the built-in exchange–correlation functional of Perdew, Burke, and Ernzerhof (PBE)^90^ was used. Scalar relativistic effects for core ionizations were accounted for using the spin-free X2C one-electron (SFX2C-1e) Hamiltonian.^43,91,92^ The reported computation times correspond to wall-clock times measured on an Intel Xeon Gold 6448H processor using 6 cores. The molecular orbitals are visualized by the IboView program.^93,94^

### 3.2 Benchmark and Representative Calculations

In the context of thermally activated delayed fluorescence (TADF) emitters used in organic light-emitting devices, the ionization energy critically influences charge injection, energy-level alignment, and operational stability.^3,95,96^ Although charge carriers traverse multiple functional layers before reaching the emissive region, the emitter IP determines the alignment within the energetic cascade formed by the surrounding transport and blocking materials. Proper alignment facilitates efficient hole transport, lowers injection barriers, and promotes balanced charge populations within the emitting layer, which is essential for maximizing quantum efficiency and ensuring optimal operation of the TADF mechanism. Conversely, an unfavorable ionization energy may lead to charge imbalance and carrier accumulation, enhancing nonradiative loss channels, such as triplet–polaron annihilation,^3^ and accelerating device degradation. Despite this importance, ionization energies are frequently approximated by the highest occupied molecular orbital (HOMO) energies, implicitly neglecting orbital relaxation and electron correlation effects, which can lead to significant deviations from the true, physically relevant ionization energies. Furthermore, accurate core ionization energies are indispensable for materials characterization by X-ray photoelectron spectroscopy (XPS).^97^

Beyond optoelectronic materials, ionization energies play a central role in biomolecular and radiation-induced processes.^6,98^ Knowledge of residue-specific IPs is essential for understanding the photoionization behavior of aromatic amino acids in proteins, as it defines the energetic threshold for charge generation under UV or X-ray irradiation.^99^ Ionization may produce radical or charged species that perturb hydrogen-bonding networks and electrostatic interactions, potentially initiating long-range electron-transfer events and secondary chemical damage. In lipid-associated systems, IPs provide essential insights into the electronic stability and radiation sensitivity of membrane components. The formation of a charged species within the hydrophobic membrane core upon ionization can disrupt local lipid packing and alter dielectric properties, thereby affecting membrane stability and charge migration.^98,100^ In particular, polyunsaturated fatty acids exhibit comparatively low ionization energies, which increase their susceptibility to photo- and radiation-induced radical formation. Such initial ionization events can trigger lipid peroxidation cascades, ultimately compromising membrane integrity and influencing cellular signaling processes.

Accordingly, the molecular systems employed in the benchmark and representative calculations were selected to reflect the above aspects. For the calculation of valence ionization energies in TADF emitters, a set of 30 representative molecules (DBCTRZ, SeDF-B, oAcTBc, PXZPyPM, Mcz-TXT, TDBA-Ac, PhlCzDP, TPAmPPC, TPA-PPDCN, 2CzdOXD4MeOPh, CzDBA, SF2-TRZ, AcCN, PxCN, HzTFEX2, CzTrz, 4CzIPN, CzPXZ, t-CzPXZ, DPmP-PXZ, CPDA-MPC, SBF-BP-DMAC, DPAC-BPI-CN, DPAC-BP-CN, DPAC-BPI, TPA-DCPP, 2CzCN, ACzCN, PhCzCN, and PzTDBA) was compiled. These emitters exhibit substantial structural diversity, encompassing a variety of donor–acceptor compounds, conjugated backbone motifs, and sterically modulated molecular systems. The structural features and key properties of most of these emitters have been reported in ref 96. From the class of biologically relevant systems, cholesterol and a 131-atom hydroxylated phosphatidylethanolamine derivative PE­(18:0/20:4­(5Z,8Z,11Z,14Z)) were chosen to probe ionization processes in membrane-associated environments.^100,101^ For the investigation of core binding energies, a benchmark set comprising 21 amino acids was employed. Nitrogen and oxygen *K*-edge ionizations were considered following ref 48, resulting in a total of 82 values included in the data set. The default cutoff parameters were further tested on the 91-atom TADF emitter PXZPyPM, which served as a larger molecular test case. As a representative large-scale application, a 372-atom porphyrin-based light-harvesting system was selected;^102^ a schematic representation of this system is shown in Figure 1. In this case, the computed core ionization energies provide access to metal coordination effects via changes in peak separations in the corresponding XPS spectra, offering direct insights into the electronic structure changes associated with metal binding.

**Figure 1:**
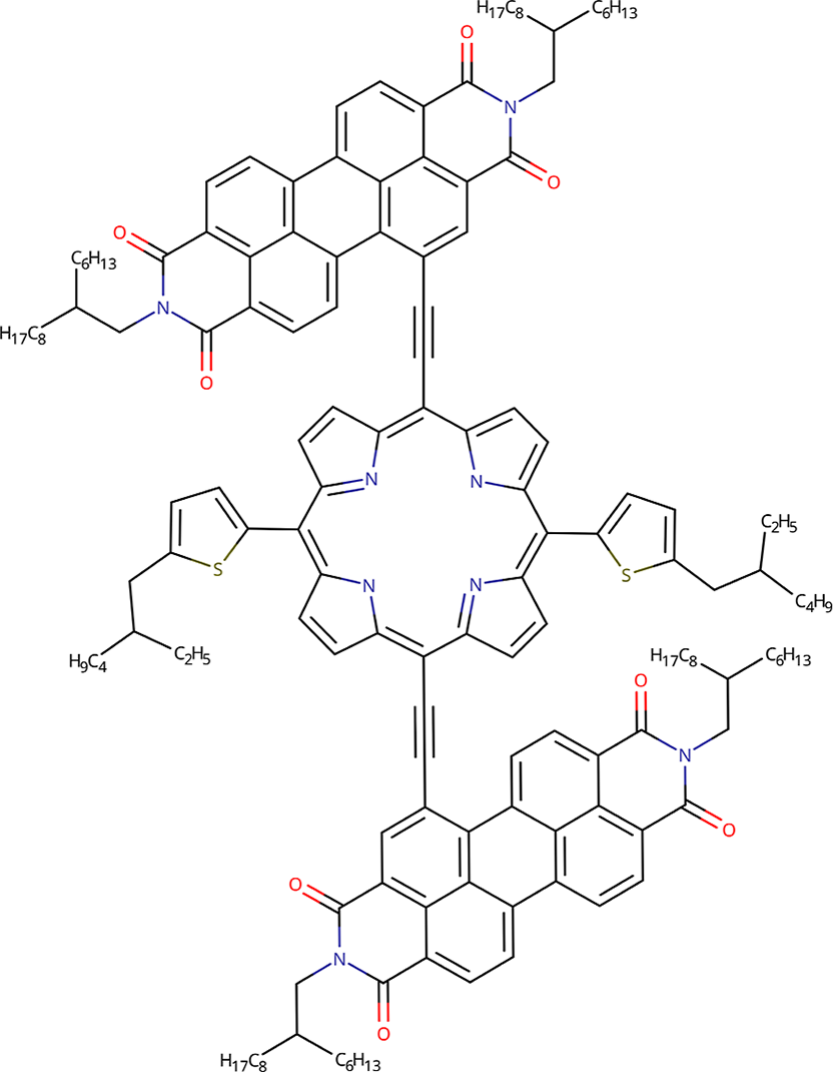
Schematic representation of the porphyrin-based light-harvesting system.

The molecular geometries of the extended systems employed in this study were obtained by using a unified protocol. First, a rapid and coarse conformational analysis was performed using CREST software^103^ in combination with the GFN-FF method.^104^ The resulting conformers were subsequently optimized using the semiempirical GFN2-xTB^105^ approach with the xtb program.^106^ From the obtained structures, the lowest energy conformer was selected for higher level calculations. This protocol was applied to all of the investigated molecules except for the amino acids. For these systems, the geometries were taken from ref 48, with the exception of histidine. The structure reported there is incorrect; therefore, the geometry of this molecule was also determined by using the protocol described above. For the higher level calculations, the basis sets described in the previous section were employed with the exception of the porphyrin derivative. In this case, the cc-pCVTZ-X2C basis set was applied to the atoms of the porphyrin ring, while the remaining atoms were described by using the cc-pVTZ basis set.

In all cases, the results obtained with the proposed framework were evaluated, relative to the corresponding canonical calculations within each method. The primary statistical measures discussed are the mean absolute error (MAE), standard deviation (SD) of the errors, and maximum absolute error (MAX). All raw numerical data, optimized geometries, and output files generated during the present study are provided in the Supporting Information.

## 4 Results and Discussion

### 4.1 Valence Ionization Energies

We first discuss the results for valence ionization energies. The violin plots summarizing the errors introduced by the local approximation in the calculated ionization energies of the TADF emitters are presented in Figure 2. Overall, the deviations remain very small for all of the investigated methods, indicating that the domain-based truncation does not compromise the accuracy of the approaches. For conventional ADC(2), the MAE amounts to 0.021 eV, with an SD of 0.026 eV and a MAX of 0.074 eV. A similar level of accuracy was obtained for SOS-ADC(2). In this case, the MAE slightly decreases to 0.016 eV, while the spread of the distribution becomes noticeably smaller, with an SD of 0.018 eV and a MAX of 0.047 eV.

**Figure 2:**
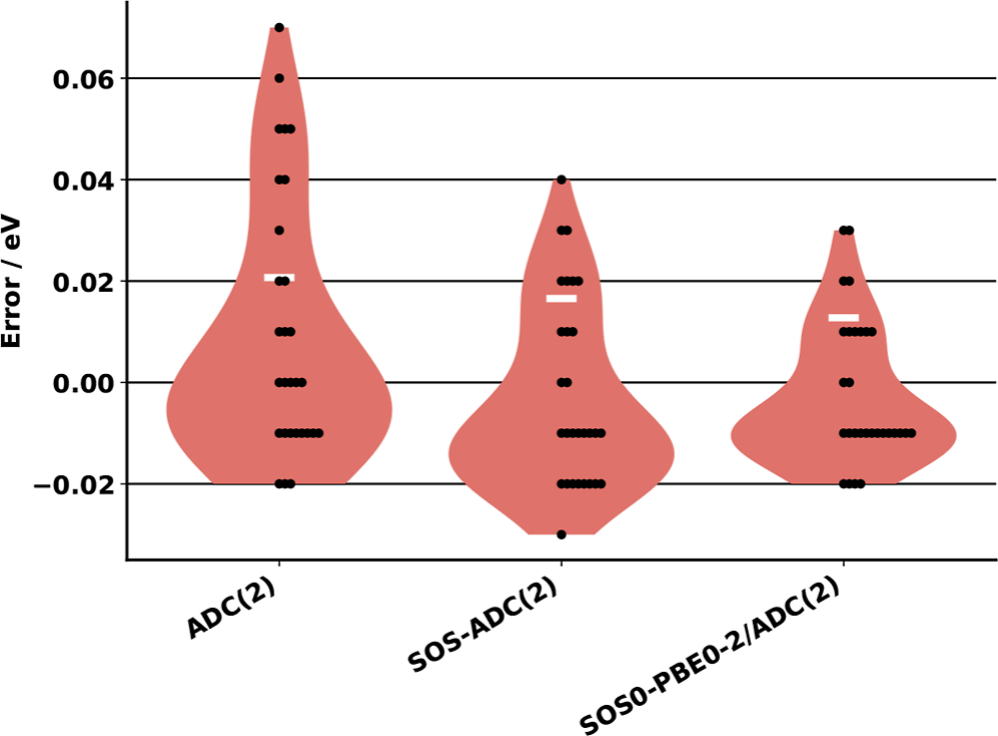
Distribution of errors introduced by the local approximation for the valence ionization energies of the TADF emitters obtained with the cc-pVTZ basis set. The white line indicates the MAE, while the black dots represent a histogram-like visualization of the error distribution.

The most compact error distribution is observed for the SOS0-PBE0-2/ADC(2) approach, which is not surprising, given that the second-order contribution entering the ADC(2) correction is scaled down in this method. Here, the MAE decreases to 0.011 eV, accompanied by an SD of 0.013 eV and a MAX of only 0.027 eV. These deviations are well below the intrinsic uncertainties of the underlying electronic structure methods, demonstrating that the errors introduced by the local approximation are practically negligible.

In addition to the accuracy of the local approximation, it is also important to assess the computational efficiency achieved with the reduced-scaling framework. In the following, we restrict the discussion to the ADC(2) results for the sake of the brevity. For SOS-ADC(2), the size of the reduced orbital subspace is identical with that obtained for ADC(2) in all cases. In contrast, for SOS0-PBE0-2/ADC(2), the constructed domains may differ slightly but typically by only a few percent due to the use of KS orbitals in the underlying reference calculation (see the Supporting Information). Consequently, the trends observed for ADC(2) can be considered to be representative for the other variants as well. The corresponding performance metrics for the investigated TADF emitters are listed in Table 1. The timing data clearly show that within the correlated part of the canonical calculations, the MP2 step is the dominant computational bottleneck in every case. Indeed, the wall-clock time required to compute the MP2 contributions is consistently at least one order of magnitude larger than that of the subsequent ADC(2) eigenvalue calculation. This is fully consistent with the formal scaling characteristics discussed above and confirms that for valence ionization problems, any substantial reduction in the overall cost must primarily originate from lowering the expense of the MP2 part.

**Table 1:** Wall-Clock Times (in min) of the Canonical ADC(2) Calculations, Retained Orbitals, and Basis Functions (in %) in the Domain, and the Resulting Speedups in the Correlated Part for the Valence Ionization Calculations of the TADF Emitters Obtained with the cc-pVTZ Basis Set

	wall-clock time				retained orbitals and functions				
molecule	HF-SCF	MP2	ADC(2)	number of atoms	AO	auxiliary	occupied	virtual	speedup
DBCTRZ	43.50	56.25	2.19	89	96.1	79.9	78.6	67.9	4.2
SeDF-B	35.23	16.89	0.62	67	100.0	100.0	100.0	92.6	1.5
oAcTBc	21.77	11.57	0.68	64	100.0	99.2	98.9	91.1	1.7
PXZPyPM	55.27	68.42	2.95	91	98.1	88.3	86.9	72.8	3.0
Mcz-TXT	65.07	45.39	1.55	89	100.0	98.9	97.5	86.0	1.9
TDBA-Ac	38.67	33.20	1.51	85	95.6	73.0	64.3	49.4	12.6
PhlCzDP	43.25	39.51	0.80	84	100.0	100.0	100.0	93.0	1.2
TPAmPPC	27.02	15.23	1.05	71	95.8	74.3	72.6	68.6	4.4
TPA-PPDCN	40.45	37.35	1.69	82	100.0	89.9	90.0	82.7	2.3
2CzdOXD4MeOPh	69.65	86.00	2.51	90	100.0	96.8	92.8	80.1	2.4
CzDBA	55.65	78.01	1.52	96	100.0	97.9	93.9	81.7	2.0
SF2-TRZ	21.68	18.54	0.63	68	94.9	73.8	72.0	58.1	9.4
AcCN	64.15	55.83	2.22	90	100.0	98.5	96.8	84.5	2.2
PxCN	26.97	23.42	0.67	74	100.0	100.0	100.0	88.0	1.3
HzTFEX2	10.97	6.04	0.48	55	100.0	86.5	79.8	80.9	3.2
CzTrz	11.65	7.21	0.34	59	94.1	69.7	67.8	60.1	8.1
4CzIPN	71.38	78.64	2.36	94	100.0	97.3	94.4	82.6	2.7
CzPXZ	19.77	13.83	0.51	66	100.0	93.2	86.1	70.8	2.9
t-CzPXZ	64.13	44.43	2.47	90	100.0	80.0	75.2	63.0	5.2
DPmP-PXZ	11.88	7.78	0.34	58	98.0	73.1	68.9	60.5	7.7
CPDA-MPC	23.83	13.37	0.58	66	100.0	95.1	91.8	82.8	2.6
SBF-BP-DMAC	50.97	37.15	1.40	82	96.4	62.1	56.0	45.8	23.6
DPAC-BPI-CN	61.23	63.56	1.55	92	99.4	84.6	81.1	69.9	4.4
DPAC-BP-CN	20.52	12.43	0.75	66	99.1	86.2	85.1	75.7	3.5
DPAC-BPI	43.83	55.97	2.13	91	99.3	84.3	80.5	67.0	4.8
TPA-DCPP	64.73	76.50	3.21	94	100.0	100.0	100.0	93.9	1.9
2CzCN	6.57	3.21	0.22	49	100.0	100.0	100.0	88.1	2.3
ACzCN	9.42	9.43	0.51	58	97.9	74.1	67.5	55.3	12.4
PhCzCN	3.40	0.70	0.09	39	100.0	100.0	100.0	84.7	2.1

At the same time, the size of the reduced orbital subspace varies strongly among systems. This behavior reflects the underlying electronic structure of the molecule, in particular, the spatial localization of the orbital that dominates the ionization process. As a consequence, the efficiency of truncation cannot be inferred from the molecular size alone. For example, for TPA-DCPP, which contains 94 atoms, only a very small reduction of the orbital spaces is achieved, indicating a comparatively delocalized ionization pattern. In contrast, for CzTrz, despite its considerably smaller size of 59 atoms, nearly half of the occupied and virtual orbitals can be neglected. This illustrates that the effectiveness of the local approximation is governed primarily by the character of the ionized state rather than by the number of atoms. In addition, no systematic correlation was observed between the truncated subspace size or system character and the magnitude of the error, further supporting the robustness of the proposed method. Importantly, the construction of the subspace does not require any user intervention. It proceeds fully automatically based on the electronic structure of the system, which makes the method applicable in a genuine black box fashion without the need for system-specific tuning or manual selection of active regions. This robustness is particularly advantageous for large and chemically diverse molecular sets where manual domain definition would be impractical.

As expected, the obtained speedups follow the extent of reduction in the retained spaces. Systems for which only minor truncation is possible exhibit comparatively modest gains, whereas strongly localized ionizations lead to much more pronounced accelerations. Nevertheless, even in the least favorable cases, the local treatment still yields a computational benefit of roughly 20% in the correlated treatment, while in several molecules, the speedup approaches or exceeds one order of magnitude. As a practical consequence, systems of the size considered here can, in most cases, be treated within wall-clock times of only a few tens of minutes using the reduced-scaling approach. This represents only a modest additional cost compared with the HF/KS-SCF calculation that is already required in standard workflows. This means a substantial practical advantage as it enables the routine evaluation of ionization energies with electron correlation methods for large molecular candidates in virtual screening workflows, where the ability to perform many calculations within short turnaround times is essential.

As a representative large-scale example, we consider the 132-atom PzTDBA molecule. The calculated ionization energies, the retained orbitals and basis functions, and the corresponding wall-clock times are summarized in Table 2. The domain construction leads to a substantial reduction of the correlated orbital space, even for this system size. While the AO space is almost fully retained, the auxiliary, occupied, and virtual spaces are reduced to a much greater extent. In particular, only ∼70% of the occupied orbitals and roughly half of the virtual orbitals remain in the domain. This reflects the spatially localized nature of the ionization process and the accompanying relaxation of the electronic structure. The computational savings associated with this truncation are clearly visible in the time data. Although the SCF step still represents a significant part of the overall cost for such a large system, the subsequent correlated treatment is comparatively inexpensive. The domain construction requires approximately 2.5 min in total. Only a negligible fraction of this time is associated with the selection of the orbitals, which can be considered an overhead. The remaining time is spent on the integral transformation, which is itself already significantly accelerated because of the truncation of the orbital spaces. The MP2 step requires less than 20 min, while the ADC(2) eigenvalue problem itself is solved within approximately 1 min. These results demonstrate that even for systems exceeding one hundred atoms, the present reduced-scaling framework enables correlated calculations of ionization energies at a computational cost that remains practical for routine applications.

**Table 2:** Ionization Energies (in eV), Retained Orbitals and Basis Functions (in %) in the Domain, and Wall-Clock Times (in min) for the 132-Atom PzTDBA Molecule Obtained with the cc-pVTZ Basis Set

		retained orbitals and functions				wall-clock time			
method	ω	AO	auxiliary	occupied	virtual	HF-SCF	domain^a^	MP2	ADC(2)
ADC(2)	5.04	98.1	68.9	67.4	49.9	183.72	2.45	18.88	0.75
SOS-ADC(2)	5.55	98.1	68.9	67.4	49.9	183.72	2.45	18.88	0.71
SOS0-PBE0-2/ADC(2)	5.60	98.1	68.9	68.5	50.5	198.30	2.52	19.53	0.77

a Domain construction including orbital selection and integral transformation.

We now turn to biomolecular systems to assess the performance of the proposed framework. The corresponding results are listed in Table 3. Overall, the deviations between the canonical and local results remain small for all the investigated methods. For cholesterol, the differences are negligible, with deviations not exceeding 0.02 eV. Slightly larger, yet still modest deviations are observed for the PE­(18:0/20:4) system, where the maximum difference remains below 0.06 eV. The observed trends are consistent with those obtained for the TADF emitters, and, as expected, the smallest deviations are found for the SOS0-PBE0-2/ADC(2) approach. At the same time, a substantial reduction of the orbital space is achieved. In both systems, the occupied and virtual spaces are reduced to roughly 45–70% of their original size, while the auxiliary basis is also significantly truncated. As a consequence, the calculations remain computationally efficient: the total wall-clock time of the correlated treatment, including the domain construction, amounts to less than 5 min for cholesterol and remains below 15 min for the 131-atom PE­(18:0/20:4) system.

**Table 3:** Ionization Energies (in eV) and Retained Orbitals and Basis Functions (in %) in the Domain for the Biomolecules Obtained with the cc-pVTZ Basis Set

		ω		retained orbitals and functions			
molecule	method	canonical	local	AO	auxiliary	occupied	virtual
cholesterol	ADC(2)	7.98	8.00	87.5	71.6	67.5	52.2
	SOS-ADC(2)	8.59	8.59	87.5	71.6	67.5	52.2
	SOS0-PBE0-2/ADC(2)	8.32	8.32	88.4	71.6	67.5	53.1
PE(18:0/20:4)	ADC(2)	7.75	7.81	85.0	56.4	50.7	45.2
	SOS-ADC(2)	8.32	8.35	85.0	56.4	50.7	45.2
	SOS0-PBE0-2/ADC(2)	8.03	8.05	85.0	60.6	55.2	47.5

An additional benefit of the present ADC(2)-based approaches is that they provide direct access to Dyson orbitals associated with the ionization process.^28^ While ionization energies are often approximated by the negative of the HOMO energy, this picture corresponds to the removal of an electron from the HOMO and therefore neglects relaxation and correlation effects. In contrast, a Dyson orbital represents the formally rigorous one-electron quantity connecting the *n*-electron ground state with the (*n* – 1)-electron ionized state, thereby incorporating the electronic response induced by the creation of the hole. Consequently, the spatial distribution of a Dyson orbital can differ significantly from that of the HOMO. An illustrative example for the 2CzdOXD4MeOPh molecule is shown in Figure 3. As can be seen, the Dyson orbital corresponding to the first ionization exhibits a somewhat more localized character than the HOMO, reflecting the local reorganization of the electron density around the ionization site and the configurational mixing that arises from the coupling of the dominant 1h configuration with additional ionization channels.

**Figure 3:**
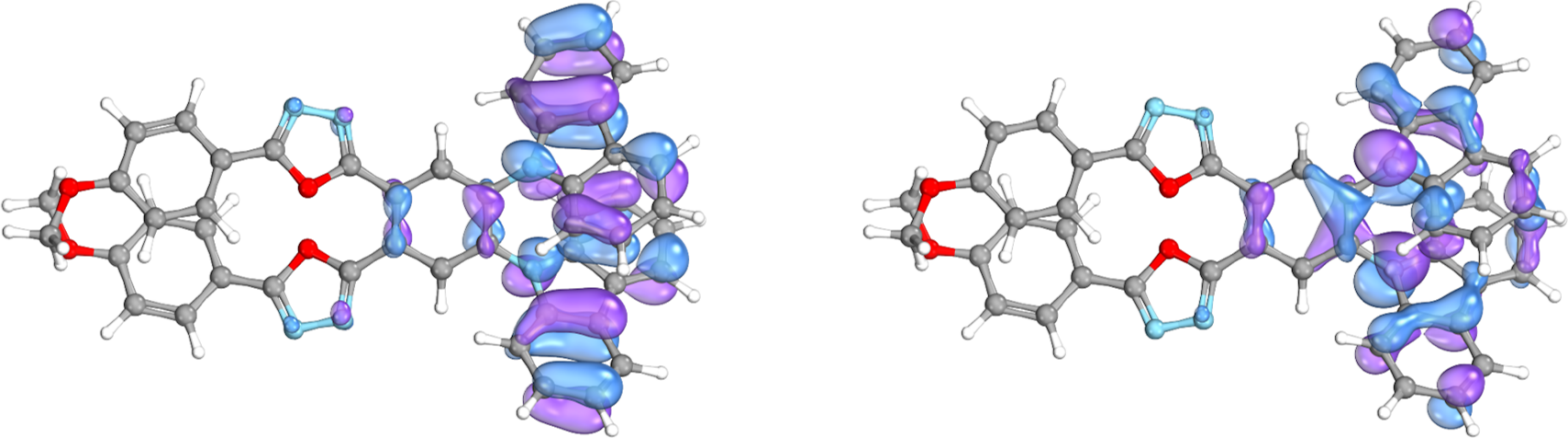
HOMO (left) and the Dyson orbital corresponding to the first ionization (right) of the 2CzdOXD4MeOPh molecule.

### 4.2 Core Ionization Energies

The distribution of errors introduced by the local approximation for the core ionization energies of the amino acids is shown in Figure 4. Overall, the deviations remain small for all of the investigated methods, indicating that the reduced domain representation provides a reliable description of core-ionized states. For the N *K*-edge ionizations, the errors are somewhat larger and more widely distributed than those for the O *K*-edge cases. For conventional ADC(2), the MAE amounts to 0.063 eV, with an SD of 0.029 eV and a MAX deviation of 0.158 eV. The accuracy improves for SOS-ADC(2), where the MAE decreases to 0.051 eV, accompanied by an SD of 0.022 eV and a MAX of 0.123 eV. The smallest deviations are obtained for the SOS0-PBE0-2/ADC(2) approach, for which the MAE is reduced to 0.030 eV, with an SD of 0.015 eV and a MAX of 0.077 eV. This behavior is fully consistent with the trends observed for valence ionization energies.

**Figure 4:**
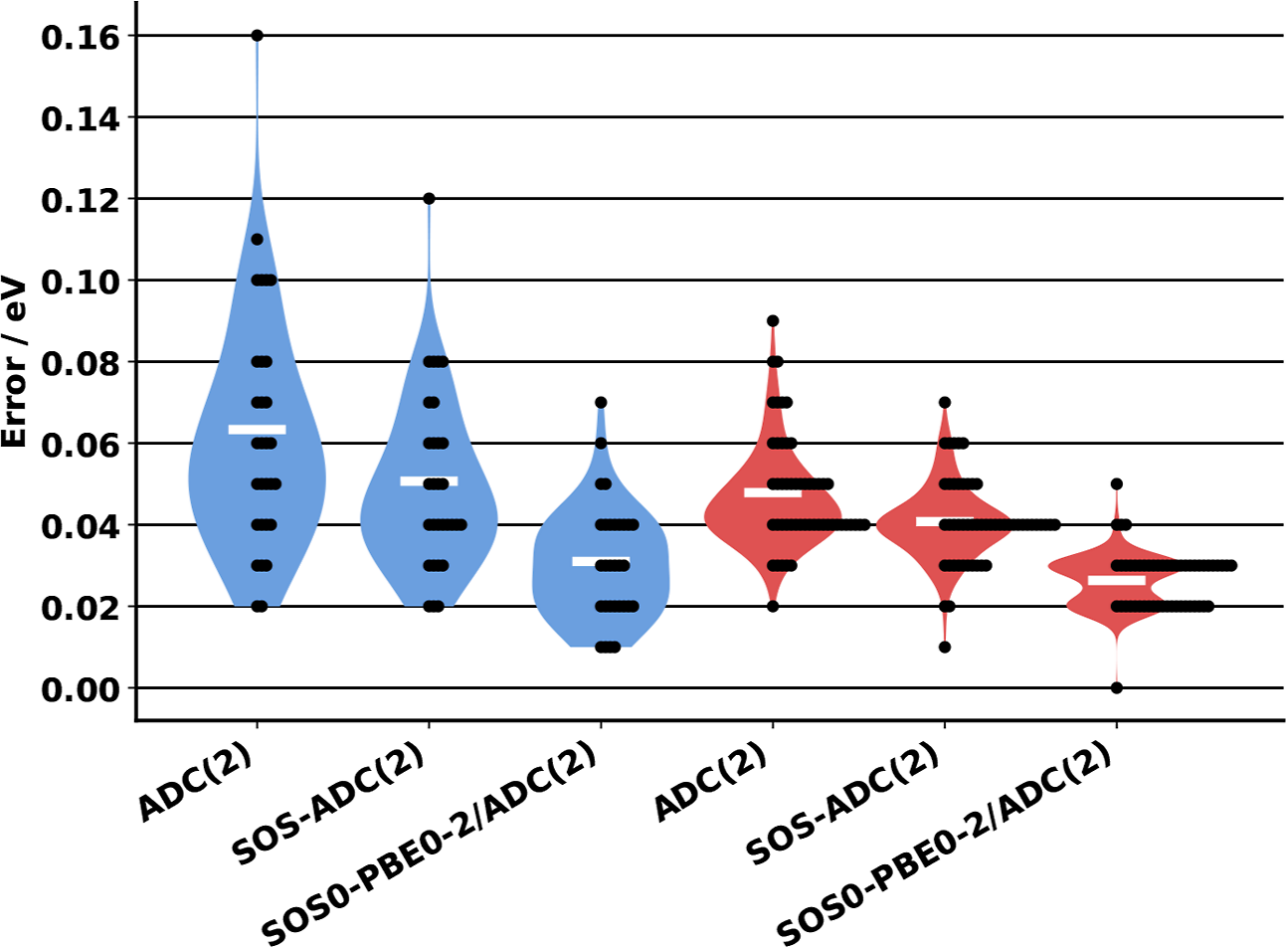
Distribution of errors for the core ionization energies of the amino acids obtained with the cc-pCVTZ-X2C basis set. The white line indicates the MAE, while the black dots represent a histogram-like visualization of the error distribution. Blue and red colors correspond to N and O *K*-edge ionizations, respectively.

A similar pattern is observed for the O *K*-edge ionizations, although the overall error level is slightly lower. In this case, the MAE amounts to 0.048 eV for ADC(2), with an SD of 0.013 eV and a MAX of 0.083 eV. For SOS-ADC(2), the MAE decreases to 0.041 eV with an SD of 0.010 eV and a MAX of 0.067 eV. Again, the most compact error distribution is observed for SOS0-PBE0-2/ADC(2), where the MAE, SD, and MAX values are reduced to 0.026, 0.008, and 0.049 eV, respectively. This is particularly encouraging, since this DH variant provided the highest accuracy and precision among the considered approaches in our latest study.^43^

At first glance, it may seem surprising that the errors are slightly smaller for the O *K*-edge ionizations, despite their higher absolute energies. However, the present data do not support a clear and general explanation for this trend, and a similar behavior has also been reported, without detailed explanation, in ref 48. To further investigate this observation, we performed an additional threshold-convergence test for the conventional ADC(2) method, shown in Figure 5, in which the domains were constructed by including all atoms, and thus all AO and auxiliary basis functions, as well as the complete virtual orbital space.

**Figure 5:**
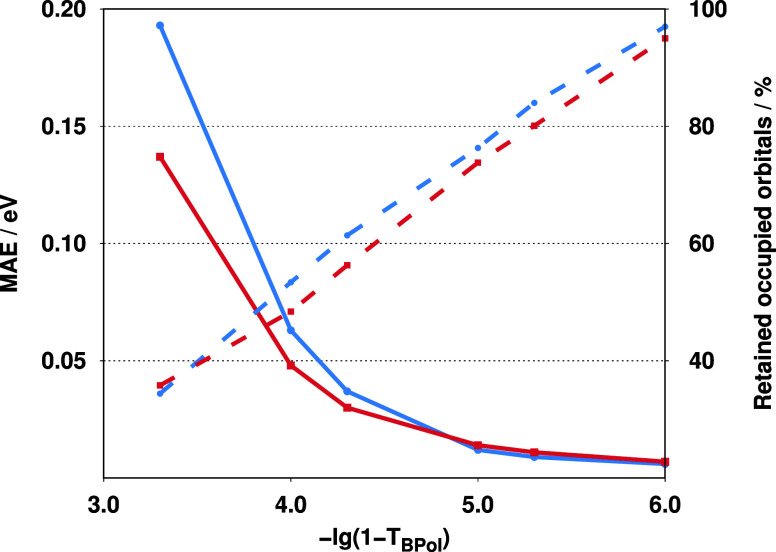
Convergence of the MAE for the N and O *K*-edge ionization energies with respect to the threshold *T*_BPol_ controlling occupied orbital selection. All atoms, AO and auxiliary basis functions, and virtual orbitals were included in the domains; only the occupied orbital space was truncated. Solid lines show the MAE, while dashed lines indicate the fraction of occupied orbitals retained in the domain. Red and blue curves correspond to O and N *K*-edge ionizations, respectively.

Only the thresholds controlling the selected occupied orbitals, *T*_BPol_ and *T*_BPot_, were varied. The current default setting is *T*_BPol_ = *T*_BPot_ = 0.9999. The *T*_BPol_ parameter was scanned between 0.9995 and 0.999999. Whenever it exceeded the value of *T*_BPot_, the latter was also increased accordingly, since it is not physically meaningful for the tight criterion to be smaller than the loose one. The MAE was unchanged upon the extension of the AO, auxiliary, and virtual space to the full system, indicating that the dominant source of the local approximation error is the truncation of the occupied orbital space rather than the selection of atoms or virtual orbitals. The results show that for the default and looser threshold values, the average error is indeed smaller for the O *K*-edge ionizations than for the N *K*-edge cases. This is particularly interesting because at the default threshold, the O *K*-edge domains retain a smaller fraction of the occupied orbitals. On the other hand, the error appears to converge marginally faster for the N *K*-edge ionizations, although this effect is relatively small. Importantly, in both cases, the MAE smoothly approaches zero as the occupied subspace is systematically enlarged, confirming that the observed deviations originate from local approximation. Nevertheless, the underlying reason for this behavior remains an open question, and the present benchmark set may be too small to draw a definitive general conclusion regarding the different behaviors of the N and O *K*-edge ionizations.

Overall, the deviations introduced by the local approximation with the default parameters remain well below 0.1 eV for the vast majority of cases and are therefore more than an order of magnitude smaller than the intrinsic errors of the underlying electronic-structure methods. This confirms that the proposed domain-based reduced-scaling framework provides a robust description of both the valence and core ionization processes.

Next, we briefly discuss the characteristics of the domains obtained for the core ionizations. It is worth noting that, in this case, a more conservative domain construction strategy was adopted. In contrast to the valence ionizations, the primary objective here is not to accelerate the ADC(2) calculation itself but rather to reduce the formal scaling and computational cost of the integral transformation, as a natural consequence of the proposed algorithm. Nevertheless, because core ionizations are extremely localized processes, the domain selection still leads to a substantial reduction of the orbital space. Despite the relatively small size of the amino acids considered here (up to 22 atoms), approximately 50% of the occupied orbitals can be discarded, while the number of virtual orbitals is reduced by about 10–20%. This can be attributed to the absence of extended aromatic delocalization in these systems. The exact values for the individual systems are provided in the Supporting Information. In both IP and CVS-IP calculations, the primary computational and memory bottleneck is associated with the occupied-virtual block of the molecular orbital integrals. Consequently, a reduction in either index dimension is equally important and provides a significant advantage. Such truncations therefore lead to noticeable gains, while at the same time, the accuracy of the underlying correlated methods remains fully preserved.

Since the amino acid benchmark set contains relatively small systems, we also tested the default cutoff parameters before turning to the large demonstration calculations. For this purpose, we selected the 91-atom PXZPyPM TADF molecule, which provides a chemically more diverse and substantially larger test case, containing benzopyran-like oxygen atoms as well as indolenine- and pyrimidine-like nitrogen centers. This test was performed for the conventional ADC(2) method for which the largest errors were observed in the benchmark calculations. The corresponding results are listed in Table 4. They show that the error obtained with the default thresholds is already very small, not exceeding 0.1 eV, even though the resulting domains involve a substantial truncation compared to the full molecular orbital space. This independent validation therefore supports the safe application of the same domain-construction protocol to the larger systems considered below.

**Table 4:** Core Ionization Energies (in eV) and Retained Orbitals and Basis Functions (in %) in the Domain for the PXZPyPM TADF Molecule Obtained at the ADC(2)/cc-pCVTZ-X2C Level

	ω		retained orbitals and functions			
atom	canonical	local	AO	auxiliary	occupied	virtual
pyrimidine-like N	404.47	404.57	93.4	95.2	23.1	77.7
indolenine-like N	405.35	405.45	72.5	73.4	21.0	54.7
benzopyran-like O	538.15	538.23	42.0	42.3	15.4	35.1

As a final representative large-scale example, we consider a porphyrin derivative containing 372 atoms together with its Zn-coordinated analogue.^102^ For the sake of brevity, we discuss here only the results obtained with the SOS0-PBE0-2/ADC(2) approach. Because of the extended system size, the KS-SCF problem was solved using a Huzinaga embedding scheme^107^ in which the porphyrin ring defines the embedded subsystem, while the remaining environment is treated at the PBE level. This strategy leads to an approximately one order of magnitude reduction in the computational cost of the SCF procedure for this system, which is particularly important since the SCF step represents the rate-determining part of the calculation. The calculated core ionization energies, retained orbital and basis functions, and corresponding wall-clock times are summarized in Table 5. Inspecting the results, we conclude that once the reference KS orbital set is obtained, the correlated treatment becomes comparatively inexpensive. The only overhead is the orbital localization and the construction of the BP atom list, which takes approximately half an hour; however, this step needs to be performed only once. The integral transformation requires about 15 min per state. In total, domain construction for each state requires roughly two orders of magnitude less time than the solution of the embedding SCF problem. Compared to a conventional SCF calculation, this corresponds to a difference of three orders of magnitude. The solution of the ADC(2) eigenvalue problem requires less than 1 min in all of the considered cases. This demonstrates that the reduced scaling framework enables the efficient treatment of large molecular systems.

**Table 5:** N *K*-Edge Ionization Energies (in eV), Retained Orbitals and Basis Functions (in %) in the Domain, and Wall-Clock Times (in min) for the 372-Atom Porphyrin Derivative and Its Zn-Coordinated Analogue Obtained with SOS0-PBE0-2/ADC(2) Using a Triple-ζ Basis Set

			retained orbitals and functions				wall-clock time		
molecule	atom	ω	AO	auxiliary	occupied	virtual	KS-SCF^a^	domain^b^	ADC(2)
porphyrin derivative	iminic N	404.89	79.6	83.2	8.1	63.55	1518.2	48.4	0.5
	iminic N	404.88	79.6	83.2	8.0	63.55		16.8	0.5
	pyrrolic N–H	407.00	79.6	83.2	7.8	63.55		16.6	0.6
	pyrrolic N–H	407.07	79.6	83.2	7.7	63.55		16.2	0.5
Zn–porphyrin derivative	Zn–N	405.44	80.3	83.8	9.1	61.22	1432.6	48.7	0.5
	Zn–N	405.45	80.3	83.8	8.9	61.22		17.1	0.6
	Zn–N	405.51	80.3	83.8	10.0	61.22		17.3	0.6
	Zn–N	405.51	80.3	83.8	10.0	61.22		17.4	0.6

a KS-SCF calculation using a Huzinaga embedding scheme.

b Domain construction including orbital selection and integral transformation.

The obtained core ionization energies reproduce the well-known spectroscopic fingerprint of porphyrin systems.^108,109^ In the nonmetalated porphyrin derivative, the two characteristic peaks associated with the iminic and pyrrolic nitrogen atoms are separated by roughly 2 eV. Upon the coordination of the Zn atom, these features collapse into a single peak corresponding to the equivalent Zn–N sites, whose energy appears between the two peaks observed in the metal-free system. This behavior is in full agreement with the textbook picture of the electronic structure changes induced by metal coordination in porphyrin complexes.

## 5 Conclusions

In this work, we have developed a scalable framework for the calculation of valence and core ionization energies within the ADC(2) formalism. The approach relies on the construction of state-specific orbital domains that exploit the spatial locality of the electronic response associated with the creation of an ionized state. The resulting domains naturally incorporate the orbitals required to describe both the primary ionization process and the accompanying relaxation of electronic structure. By restricting the correlated treatment to compact orbital subspaces, the computational cost can be substantially reduced without compromising the accuracy of the resulting ionization energies. Because the domain construction is driven entirely by the underlying electronic structure, the procedure adapts automatically to the character of the ionized state and can therefore be applied in a genuine black box fashion without system-specific tuning.

Extensive benchmark calculations demonstrated that the errors introduced by the local approximation are very small. For the valence ionization energies, the deviations remain on the order of a few hundredths of an electronvolt for all of the considered ADC(2)-based variants. Similarly, small errors are obtained for the core ionization energies of the amino acids, where the deviations remain well below 0.1 eV in the vast majority of cases. These values are more than an order of magnitude smaller than the intrinsic uncertainties of the underlying correlated methods, confirming that the proposed domain construction preserves the accuracy of canonical approaches.

Finally, the applicability of the proposed framework was demonstrated for large-molecular systems. For valence ionizations, calculations on a TADF emitter containing more than 130 atoms show that substantial reductions of the correlated orbital space can be achieved, leading to a significant acceleration of the MP2 step, which represents the dominant computational bottleneck in IP-ADC(2) calculations. Even for systems of this size, the MP2 contributions can be evaluated within approximately 20 min, while the subsequent IP-ADC(2) eigenvalue problem requires only about 1 min. For core ionizations, the method was applied to a porphyrin derivative containing more than 370 atoms. In this case, the SCF procedure represents the rate-determining step and remains significantly more expensive than the subsequent correlated treatment even when accelerated using an embedding scheme. Once the reference orbital set is obtained, the correlated treatment, including the domain construction and the associated integral transformation, requires roughly two orders of magnitude less time than the embedding SCF calculation. The CVS-IP-ADC(2) eigenvalue problem itself still takes only about 1 min per state and accurately reproduces the characteristic spectroscopic signature associated with metal coordination in porphyrin systems.

These results demonstrate that the present reduced-scaling approach enables the routine application of correlated methods to molecular systems that would otherwise be inaccessible with conventional implementations.

## Supplementary Material





## Notes

Calculated ionization energies and fractions of retained orbitals and functions (XLSX)
Output text files and optimized geometries (ZIP)
